# Measurement of Pressure Fluctuations inside a Model Thrust Bearing Using PVDF Sensors

**DOI:** 10.3390/s17040878

**Published:** 2017-04-16

**Authors:** Andrew Youssef, David Matthews, Andrew Guzzomi, Jie Pan

**Affiliations:** School of Mechanical and Chemical Engineering, The University of Western Australia, 35 Stirling Hwy, Crawley, WA 6009, Australia; andrew.youssef@research.uwa.edu.au (A.Y.); david.matthews@dsto.defence.gov.au (D.M.); andrew.guzzomi@uwa.edu.au (A.G.)

**Keywords:** PVDF, thrust bearing, condition monitoring, 3D printing

## Abstract

Thrust bearings play a vital role in propulsion systems. They rely on a thin layer of oil being trapped between rotating surfaces to produce a low friction interface. The “quality” of this bearing affects many things from noise transmission to the ultimate catastrophic failure of the bearing itself. As a result, the direct measure of the forces and vibrations within the oil filled interface would be very desirable and would give an indication of the condition of the bearing in situ. The thickness of the oil film is, however, very small and conventional vibration sensors are too cumbersome to use in this confined space. This paper solves this problem by using a piezoelectric polymer film made from Polyvinylidine Fluoride (PVDF). These films are very thin (50 μm) and flexible and easy to install in awkward spaces such as the inside of a thrust bearing. A model thrust bearing was constructed using a 3D printer and PVDF films inserted into the base of the bearing. In doing so, it was possible to directly measure the force fluctuations due to the rotating pads and investigate various properties of the thrust bearing itself.

## 1. Introduction

Thrust bearings are an important component in many mechanical systems. They permit rotational motion under axial force while trying to minimize friction. There are several different types of thrust bearings such as cylindrical thrust bearings, magnetic thrust bearings and fluid filled thrust bearings. The tilted pad thrust bearing, developed by Michell and Kingsbury [1] more than 100 years ago, in its simplest form is comprised of pads at a fixed angle. More sophisticated designs allow the pads to automatically adjust their angle to suit the operating conditions. Regardless of the bearing type, fluid filled bearings all rely on the axial load being supported by a thin layer of oil.

Clearly, deterioration of the bearing can lead to catastrophic results and the ability to accurately monitor the health of the bearing is desirable. Although this can be achieved to some degree using conventional ceramic or Microelectromechanical systems (MEMS) transducers, their physical size and properties make it difficult to install them in confined spaces. As a result, they tend to be attached to the outside of the object of interest and the condition inside inferred. This paper attempts to address this problem by using a polymer piezoelectric film called Polyvinylidene Fluoride (PVDF), which can be installed inside the bearing to directly measure the forces developed in the oil film itself.

Piezoelectricity was discovered in PVDF by Kawai in 1969 [2] and since then has been used for a number of applications from underwater acoustics [3], medical imaging [4], modal analysis [5] and space applications [6].

The use of PVDF sensors for sensing capabilities is nothing new [7,8,9,10]; however, their applications have typically been limited to measuring the pressure of gases, as the strain of the PVDF can be linked with the pressure applied to the sensor. Little literature was found in the measurement of the contact force between the stationary and moving parts of a thrust bearing. Kim et al. [11] have reported on using PVDF to measure support loads in elastomeric bearings, but these were stationary rubber mounts. A similar application has been discussed by Talbot [12], who imbedded sensors in a stationary rubber material in order to monitor dynamic loads. Grinspan [13] was able to achieve the measurement of the contact force of a water droplet with a solid surface and compared this with the droplet of oil. It has also been reported that PVDF film has been embedded inside the insole of a shoe to measure the contact force [14]; however, these applications only observe a single force that is normal to the plane of the PVDF film.

PVDF has many properties that make it a potential candidate to use in order to directly measure the loads inside an operational thrust bearing [15]. Apart from condition monitoring, it may also be possible to measure other properties of the bearing such as the hydrodynamic stiffness of the oil film. This, in turn, may lead to a better understanding of the vibrations transmitted through the bearing and result in new methods in reducing the induced structural vibration through the bearing. The authors of this paper have previously published preliminary results of using PVDF sensors in rotating bearings [16]; however, a detailed analysis and mathematical model of the contact force were missing. This paper addresses this by introducing a mathematical model that describes the interaction between the thrust bearing pads and oil, and this model is also used to characterise some of the features seen in the signature obtained.

## 2. Materials and Methods

### 2.1. Experimental Setup

**Figure 1:**
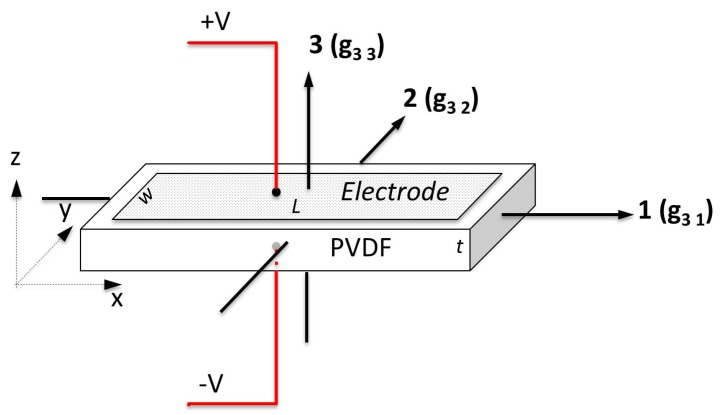
PVDF sensitivity directions.

Shielded DT1-028K type Polyvinylidene Fluoride (PVDF) films (Measurement Specialties, Hampton, VA, USA) were used to measure the interaction between the moving and stationary parts of the bearing. These are shielded piezoelectric sensors with an active area of 30 mm × 16 mm and come with cables attached. They are fabricated from DT1 films by doubling the film over in order that the outer electrode acts as an electromagnetic shield. Consequently, the original size of the film needs to be 60 mm × 16 mm × 28 μm in order to end up with the final SDT1 dimensions. When doubled over, the film thickness is effectively 56 μm. The DT1 sensors have a quoted sensitivity of 14.4 V/N in the $g_{31}$ direction, 0.013 V/N in the $g_{33}$ direction and $g_{32}$ is reported as negligible [15], where the directions are shown in Figure 1.

**Figure 2:**
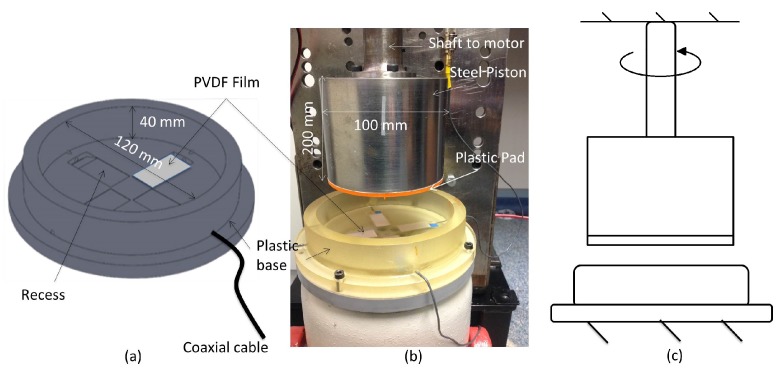
(**a**) the plastic base with the built in PVDF sensors and (**b**) the entire bearing including the upper rotating piston with interchangeable disks (orange), (**c**) schematic of the system.

Figure 2a shows the model thrust bearing. It consists of a cylindrical base of diameter 120 mm and height 40 mm and was constructed out of Stratasys 720 Full Cure ink (Stratasys, Eden Prairie, MN, USA) using a 3D printer. Four equally spaced rectangular recesses (30 mm × 20 mm × 2 mm) were generated through the printing process in the base to mount the PVDF sensors. Each sensor was carefully attached to the base using cyanoacrylate glue. The sensor wires were fed through a hole in the side of the plastic base and a thin layer of epoxy poured over the top to cover the sensors, sufficient to make the surface flush with the base. In order to minimize electromagnetic noise, Subminiature Version B (SMB) connectors were attached to the end of the coaxial wires.

In a conventional thrust bearing arrangement, the pads remain at rest and the opposite flat surface rotates. However, imbedding the sensors in the rotating face of the bearing adds additional complexity to the design of the system, and it was decided to simplify the build by locating the PVDF films in the flat stationary base and attaching the interchangeable pads to the upper rotating face. The relative motion of the pads to the PVDF is the same for both conditions.

The sensitivity of individual films encased in the base was tested using a PCB Piezotronics 086C01 impact hammer (PCB Piezotronics, Depew, NY, USA). Each film was subjected to a force perpendicular to the large face of the film ($g_{33}$ direction shown in Figure 2) and the corresponding output voltage recorded. This gave a good indication of the variability between individual films. In the model thrust bearing, however, the induced forces are three-dimensional with all three piezoelectric constants contributing to the output voltage of the PVDF film. As a result, it is difficult to use the impact hammer results to infer the absolute values of the pressures inside the oil filled bearing, as only a single direction is being excited. This is not considered an issue as the combination of forces contribute to the absolute response of the system, which tells us about the interactions between the pads and the oil. Work is currently being undertaken to derive the absolute pressures of the system. With this, the potential for these sensors in these applications is great.

**Figure 3:**
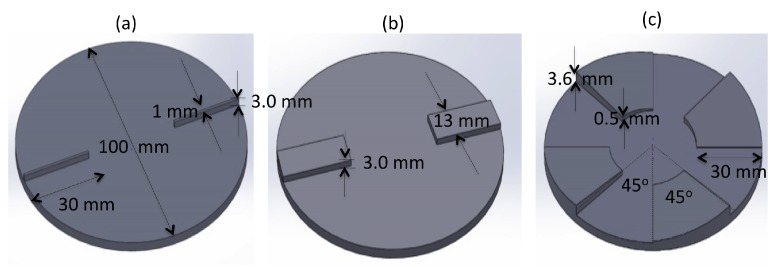
Drawings of the three interchangeable disks. (**a**) shows the 1 mm × 3 mm × 30 mm wall, (**b**) the 13 mm × 3 mm by 30 mm wall and (**c**) the 5° angled pads.

Figure 2b is a photo of the complete test rig, and the top half of the bearing consists of a steel cylinder of diameter 100 mm and height 200 mm. This was directly coupled to a DC motor that was mounted to a heavy steel plate, and a schematic of the system is shown in Figure 2c. On the bottom face of the steel cylinder, a thin interchangeable plastic disc was attached using double-sided tape (orange line in Figure 2b). This disc represents the upper face of the thrust bearing where the pads are located. Various pad shapes could easily be fabricated using a 3D printer, and, for this report, three specific designs were investigated: a 1 mm step pad, a 13 mm step pad and an angled pad. The dimension of each pad is shown in Figure 3.

The steel cylinder was positioned concentrically in the plastic base above the PVDF films. Thin metal shims (600 μm) were placed under the base until both faces were touching and gradually removed to adjust the height from 600 μm to 3600 μm at 600 μm increments. Engine oil of SAE 80w 90 viscosity was then poured into the base to a suitable level, and 80 w 90 viscosity oil is a relatively thick oil that is typically found in thrust bearings [17]. The upper cylinder was rotated at speeds from 0 to 300 rpm at 20 rpm increments, and the output voltage of the PVDF films were recorded. The rotational speed was measured using a laser tachometer and reflective tape that was aligned with the leading edge of one of the films.

The output voltage from the PVDF films was conditioned using a Brüel and Kjaer Type 2635 charge amplifier (Brüel and Kjaer, Nærum, Denmark) using a gain of zero dB, a lower frequency limit of 1 Hz and an upper frequency limit of 30 kHz. The output signals together with the laser tachometer signal were digitized using a Brüel and Kjaer 3050-B-060 data acquisition unit with a bandwidth of 6.4 kHz. Data was recorded for 60 s for each run. A filter was used to remove electromagnetic noise of 50 Hz, and this was done by applying an ideal low pass filter at 48 Hz in MATLAB (Version 2012a, Mathworks, Natick, MA, USA), and the signature that was generated by the pads over the PVDF would have occurred at the pad passing frequency of 20 Hz.

### 2.2. Mathematical Model

To gain a better understanding of the pad signatures, a mathematical model was developed based on Bernoulli’s equations [18] to approximate the pressure seen around the pads. For incompressible flow and considering that the viscous loss in the small region is small, Equation (1) is used:

(1) $$\;{\frac{V_{1}}{2}}^{2}+\frac{P_{1}}{\rho }={\frac{V_{2}}{2}}^{2}+\frac{P_{2}}{\rho }.$$

**Figure 4:**
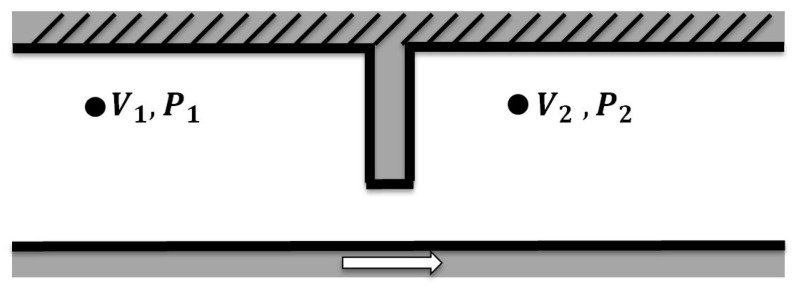
Schematic of the system in question.

This equation is applied to the system shown in the Figure 4. Because the oil is being drawn along by the pad, the inflow $V_{1}$ is traveling slower than the outward flow $V_{2}$, and the oil will then slow down due to the viscosity until the next pad approaches. $P_{1}$ and $P_{2}$ represent the pressures at positions 1 and 2, respectively. ρ is defined as the density of the fluid—in this case, oil.

It seems reasonable to assume that the velocity profile will have the following attributes:

1. The inflow velocity is slower than the outflow i.e., $V_{1}$ < $V_{2}$ (as discussed above).
2. The velocity around the step and the top wall is zero (due to the no slip condition).
3. The velocity of the bottom surface is the velocity of rotation (due to the no slip condition).
4. The velocity that passes underneath the step is at the same velocity of rotation.

For the purpose of predicting the general features of the waveform, variations in oil thickness have been ignored as well as oil flow radially in the bearing making this a 2D solution.

With regards to the tilted pad, the pressure formed underneath the tilted pad can be predicted using the well-established Reynolds equation [17]. However, in order to describe the pressure variations in front and behind the pads, it is necessary to use Bernoulli’s equation. The one-dimensional Reynolds equation where the “long bearing approximation” is used is shown below:

(2) $$\;\frac{dP}{dx}=6U\eta \frac{h-\bar{h}}{h^{3}},$$

where *P* is the pressure, *x* is the dimension along the bearing, *U* is the sliding speed η is the viscosity of the oil, *h* is the oil film height and $\bar{h}$ is the oil film height where the maximum pressure is observed i.e., $\frac{dP}{dx}=0$. The geometry of the tilted pad is expressed by Equation (3):

(3) $$\;h=h_{o}\left(1+\frac{Kx}{B}\right),$$

where $h_{o}$ is the outlet film height, *K* is defined as the convergence ratio of the bearing and *B* is the length of the bearing. Using the boundary conditions, it can be shown that $\bar{h}$ can be substituted for $\frac{2h_{o}h_{i}}{h_{i}+h_{o}},$ where $h_{i}$ is the inlet oil film thickness. Using the above equations and the assumption that the pressure at the inlet of the bearing and outlet of the bearing is equal to zero, it can be shown [17] that the pressure can be described by the following relationship:

(4) $$\;P=\frac{6U\eta B}{Kh_{0}}\left(-\frac{1}{h}+\frac{h_{o}}{h^{2}}\frac{\left(K+1\right)}{\left(K+2\right)}+\frac{1}{h_{o}\left(K+2\right)}\right).$$

A detailed description of the derivation can be found in [17]. The force applied to the PVDF was calculated by integrating over the specified film geometry. The assumption to use a ‘long bearing approximation’ (oil does not flow transverse to the direction of motion of the pad) may not necessarily be true for our application due to the size of the pad and the circular arrangement of the bearing as shown in Figure 3. However, it has been shown that the effect of this leakage is to move the position of the maximum pressure closer to the outlet of the pad with the overall shape remaining the same [19].

## 3. Results and Discussion

**Figure 5:**
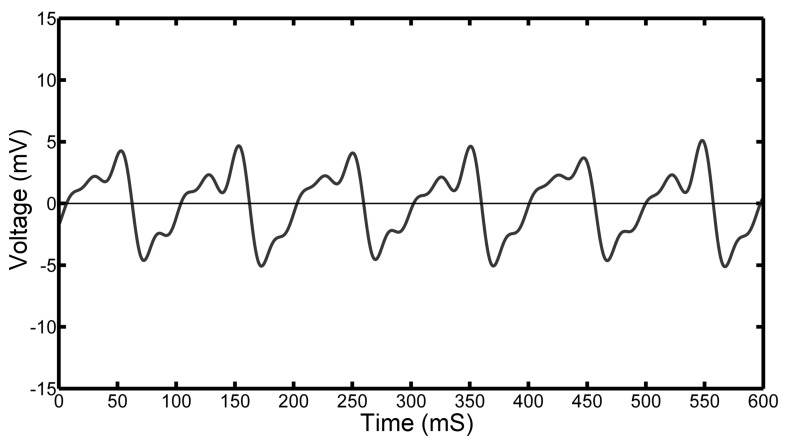
Typical voltage output from the 1 mm pad rotating at 300 rpm. Two distinct peak features can be observed corresponding to the two separate pads on opposite sides of the bearing.

Figure 5 shows a typical time dependent voltage from one PVDF film for several revolutions of the 1 mm pad. Two distinct sets of peaks are observed corresponding to the two individual pads as they pass over one PVDF sensor. The difference in shape and amplitude of the two peaks is due to small misalignments of the pads introduced when setting up the equipment. As can be seen, both positive and negative voltages can be observed corresponding to regions of positive and negative pressures. In addition, the resulting shape is quite complex with a number of inflection points observed between the two main peaks. In order to gain better understanding into the mechanism responsible for this shape, the positions of the pads relative to the underlying PVDF film were accurately monitored using a laser tachometer while measuring the voltage output from the film.

**Figure 6:**
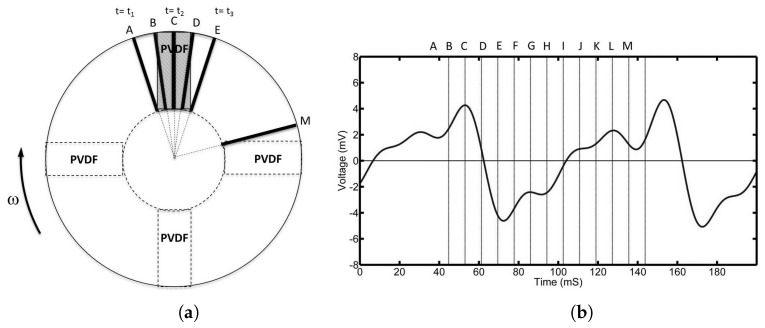
(**a**) shows the position of the 1 mm pad relative to the top PVDF film (A to E). The corresponding voltage output is shown in (**b**).

Figure 6b shows the output signal for the 1 mm pad over a time period of 200 ms. This corresponds to half a revolution of the pads at a speed of 300 rpm. The height of the pads above the PVDF film was kept constant at 0.5 mm.

The position of the front edge of the pad (black rectangles) relative to the PVDF film is shown in Figure 6a. Positions A to E correspond to the region where the pad has direct interaction with the underlying film. As can be seen, once the pad starts to pass over the film (A), the voltage starts to increase. This voltage has a maximum at position B when the top edge of the pad crosses the film. Beyond this point, the voltage gradually decreases to zero at position C, which lies approximately half way across the film. The voltage then continues to decrease to a minimum value between D and E and then starts to increase again until it passes through zero at point H, which is approximately midway between the films. Between E and M, a number of inflection points can be observed until the process is repeated for the second pad.

**Figure 7:**
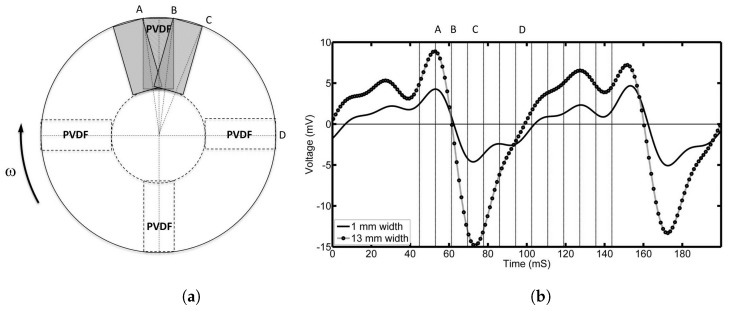
(**a**) shows the position of the 13 mm pad relative to the top PVDF film (A to C). The corresponding voltage output is shown in (**b**) (grey curve). For comparison, the output of the 1 mm step pad has been shown in black.

Similar data was also recorded for the 13 mm step pad and is shown in Figure 7. Figure 7b shows the output voltage for half a revolution. Figure 7a shows the position of the 13 mm pad relative to the top PVDF film. Voltages at times A, B, C and D correspond to the positions A, B, C and D in Figure 7a. For comparison, the voltage response for the 1 mm step pad has also been shown in black. It is clear that the maximum and minimum voltage occur at the same positions for both pads regardless of length. The voltage produced when the pad is between the PVDF films also has a similar shape. It should be noted that, for the 13 mm pad, the negative voltages are almost double that of the positive, indicating a larger negative pressure behind the pad.

**Figure 8:**
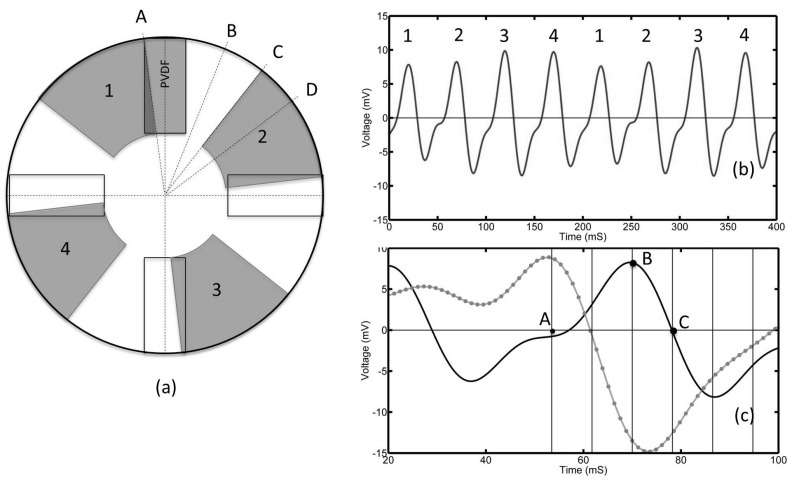
(**a**) shows the relative position of the pads (in red) to the PVDF sensors (dashed rectangles). The time dependent voltage output is shown in (**b**) and comparison between the step pads is shown in (**c**).

Figure 8 shows the results for the tilted pad. The voltage output from the top PVDF film is shown in Figure 8b. In addition, 1, 2, 3 and 4 correspond to the four separate pads.

While the voltage output shape appears similar to those obtained for the step pads, closer inspection shows that the maximum and minimum voltages occur at different pad positions than those shown in Figure 6 and Figure 7. Zero voltage occurs when the position of the top of the pad has just crossed over the PVDF film. This corresponded to a maximum voltage for both of the step pads. A maximum voltage is generated when the pad is directly over the film in position B. In position C, the pad is just starting to leave the film and zero voltage is observed. A minimum voltage is seen in position D when the pad has left the film. For comparison, the corresponding output of the 13 mm step pad has also been shown in Figure 8c in grey.

**Figure 9:**
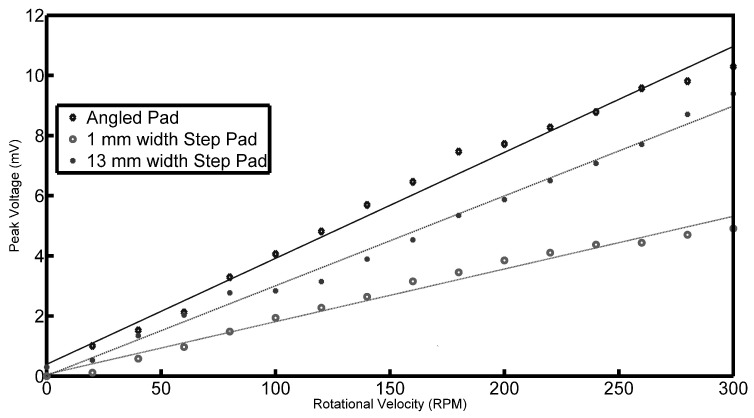
Peak voltage as a function of oil film shear rate.

As would be explained and as can be seen from Equation (4), the pressure under the tilted pad should be directly proportional to the speed of the pad. To verify this, the PVDF output voltage was monitored as a function of speed from 0 to 300 rpm in increments of 20 rpm. It was found that the overall shape of the waveform remained constant; however, the amplitude of the signal increased with increasing speed. Figure 9 shows the peak voltages observed by the PVDF film against the speed for all three pads. The black line represents the angled pad and clearly shows that the pressures generated by the sliding pads is a linear function of speed as predicted by Equation (4). Fitting a linear regression line results in a coefficient of determination $R^{2}$ of 0.9879 to 0.9977, meaning that a strong linear relationship is present. The two circle markers shows similar results for the two step pads. It is interesting to note that these also show a linear variation with speed except with smaller gradients compared to the tilted pad.

**Figure 10:**
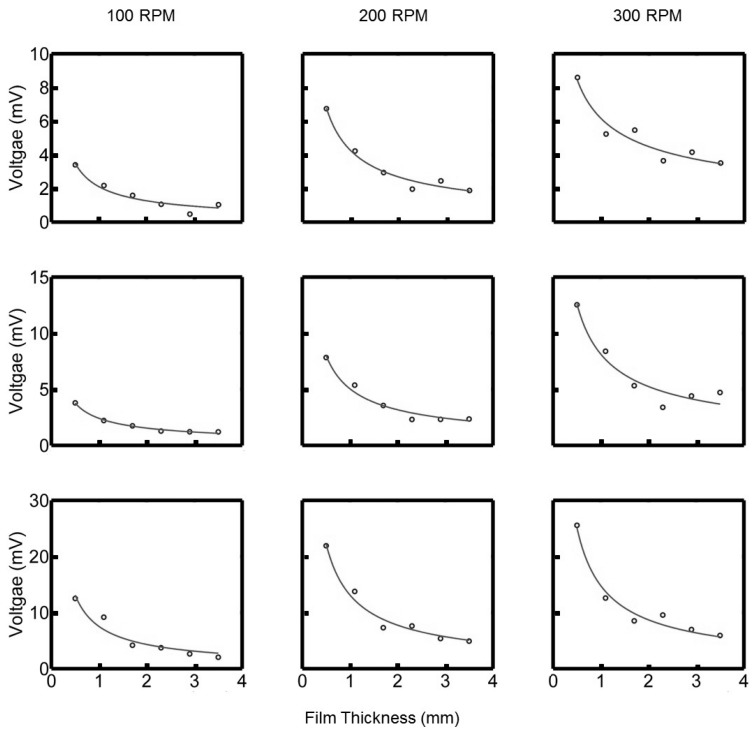
Peak voltage as a function of oil film shear rate, where (top) and (middle) is the 1 and 13 mm stepped pad respectively and (bottom) is the tilted pad.

A similar investigation was done for the height variation of the oil film. The thickness of the oil film was increased from 0.6 mm to 3.6 mm in increments of 0.6 mm. These results are shown in Figure 10 for the three rotational speeds 100, 200 and 300 rpm. The output voltage is inversely proportional to the oil film thickness *h*. The lines represent the logistic function that is fitted to the data.

**Figure 11:**
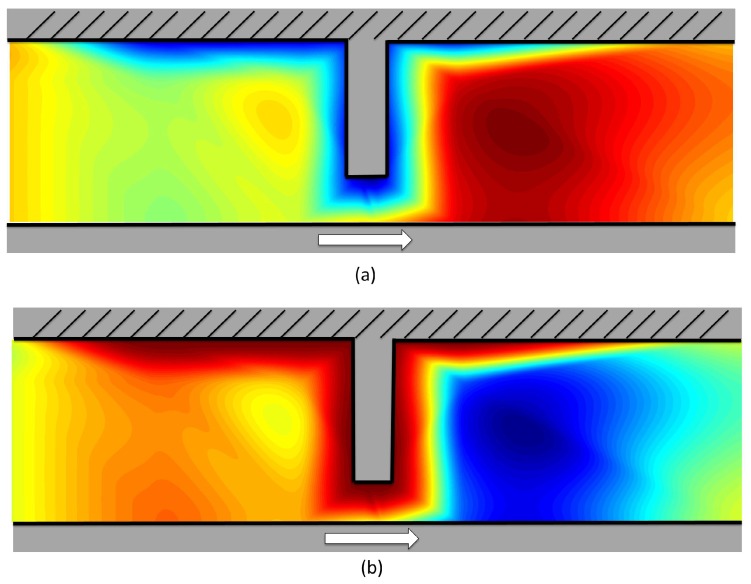
(**a**) assumed initial velocity profile generated by Equation (1); (**b**) calculated pressure field.

The mathematical models presented in Section 2.2 were used to predict the force characteristics due to the tilted pad. Comparisons of absolute pressure values were not possible due to the anisotropy issues of the calibration of the PVDF film discussed above. An initial velocity profile was generated using the boundary condition described in Section 2.2 and is shown in Figure 11a. Red represents high velocity and blue zero velocity. The orange region on the left-hand side corresponds to the inflow, while the red region is the outflow.

Using this velocity profile, the pressure at different locations was calculated using Bernoulli’s equations, assuming that the inflow pressure is zero. The result is shown in Figure 11b. Green, red and blue indicate zero pressure, positive pressures and negative pressures, respectively.

**Figure 12:**
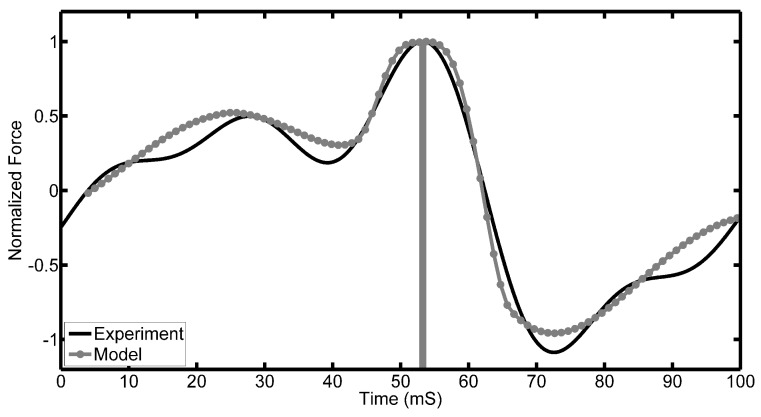
Experimental and simulation results of 1 mm stepped pad. The time at which the pad interacts with the PVDF is shaded.

Figure 11 clearly shows that, when the velocity of the oil is accelerated (+ve and −ve) by the step, it generates pockets of positive and negative pressures in front and behind the pad, respectively. The sensors’ response, however, is the sum of applied pressure across the entire film, and, as a result, the film output can be obtained by integrating over the length of the sensor. The result for the 1 mm pad is shown in Figure 12 in red. The black curve is the experimental data. Both data sets have been normalised to help comparison. As can be seen, there is good agreement, especially in the region when the pad interacts with the film. The small inflection points observed on either side of the main peak in the experimental data may be due to oil movement in the radial direction. In the model, it is assumed that the surfaces have infinite width in the direction perpendicular to the motion, and, as a result, no flow is possible in this radial direction.

A feature to note is that the maximum force observed is just before the leading edge of the step, as shown in Figure 12. This is where the oil comes to a complete stop. Past this point, the oil accelerates to the max velocity, resulting in a negative pressure. After this, the oil begins to slow down, equalizing the pressure.

Similar analysis can be done for the tilted pad. However, in order to do this, it is necessary to couple the Reynold and Bernoulli models to give an approximate prediction of the pressure wave in front, under and behind the pad. It is necessary to couple the two models at the leading and trailing edge of the pads because zero pressures are expected at the inlet and outlet of the bearing.

**Figure 13:**
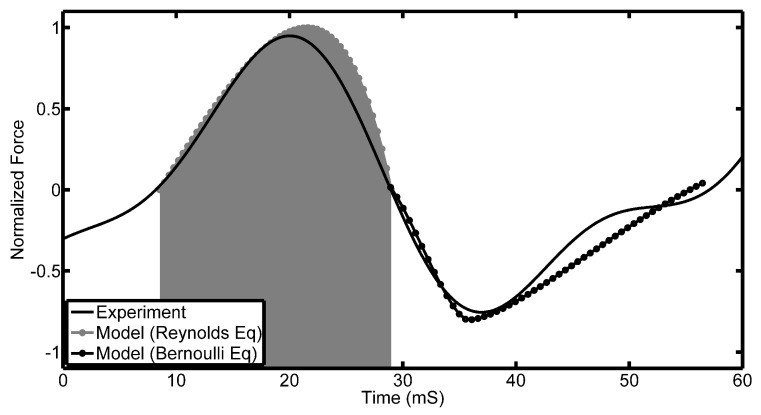
Experimental and simulation results of tilted pad. The time at which the pad interacts with the PVDF is shaded.

Figure 13 shows the normalised force as a function of time for the tilted pad. The grey curves show the theoretical results using the Reynolds and Bernoulli equations over their respective domains. The black curve is the experimental result. From the results, it can be seen that:

- At the inlet of the pad entering and after the outlet leaving the PVDF, the measured force is zero. This agrees with the assumptions made above.
- A maximum force is observed by the PVDF directly underneath the bearing. The position of this maximum force lies closer to the outlet of the bearing. This is more prominent in the theoretical result that assumes a pad of infinite width. This observation is also explained by the assumption made earlier when describing Reynolds equation.
- The inflection point observed experimentally after the main peak is not present theoretically. As mentioned above, this is most probably due to infinite pad approximation.

The mechanism of the oil force observed under the tilted pad is through the generation of a viscous liquid film between the moving surfaces. Since the top surface is inclined at a certain angle to the bottom surface, a pressure field is generated; otherwise, there would be more lubricant entering the wedge than leaving it due to the smaller cross-sectional area. In addition to further describing the waveforms of the signatures, the model can also assist in explaining the speed and height variations. In Figure 9, it was shown that there is a linear relationship between the observed voltage peaks and the speed of rotation. This observation is in line with what is expected, and Equation (4) describes the pressure field that exists underneath a tilted pad, and it is clear that the pressure field is directly proportional to speed. Concerning the height variation of the oil film, this is not as easy as describing using the presented equation. In Figure 13, it is clear that the relationship between the oil film height and the pressures observed are not directly proportional, but rather inversely proportional.

## 4. Conclusions

It has been shown that by embedding thin PVDF piezoelectric films into the base of a model thrust bearing, it is possible to measure the pressure fluctuations in an operational thrust bearing. By using a 3D printer, it was easy to investigate the effects of different pad shapes on the internal pressure fluctuations. The results were easily explained using Bernoulli and Reynolds equations, indicating that the PVDF was measuring the real pressure fluctuations inside the bearing. It was not possible, however, to get absolute values for the experimental pressures due to the anisotropic nature of the PVDF, which made calibration difficult. Future work involves building a conventional thrust bearing with intrinsic PVDF sensors and testing its performance in a working system. In addition to this, investigating thinner oil films should be investigated.
